# The high cost of celiac disease in an Israeli Health Maintenance Organization

**DOI:** 10.1186/2191-1991-3-23

**Published:** 2013-11-07

**Authors:** Anthony D Heymann, Moshe Leshno, Ronit Endevelt, Raanan Shamir

**Affiliations:** 1 The Medical Division, Maccabi Healthcare Services, Tel Aviv, Israel; 2 Sackler Faculty of Medicine, Tel Aviv University, Tel Aviv, Israel; 3 School of Public health University of Haifa, Haifa, Israel; 4 Institute for Gastroenterology, Nutrition and Liver Diseases, Schneider Children's Medical Center of Israel, Tel Aviv, Israel; 5 Faculty of Management, Tel Aviv University, Tel Aviv, Israel

**Keywords:** Celiac disease, Costs and cost analysis, Health Maintenance Organization

## Abstract

**Background:**

The aim of this study was to identify costs in patients diagnosed with Celiac disease.

**Methods:**

This retrospective case control study covered the period 2003–2006 and was conducted in a large Israeli Health Maintenance Organization insuring over two million members. Our cohort comprised 1,754 patients with Celiac disease with a control group of 15,040. Costs were aggregated according to main cost-branches and computed individually for each member. A linear step wise regression was performed with costs being the dependent variable and the independent variables; age, gender and the presence of celiac disease. Costs were compared with patients suffering from other chronic diseases.

**Results:**

The total costs of the patients with celiac disease were significantly higher than that of the control group for hospital admission, medications, laboratory and imaging. Hospital admission rate was 7.98% as opposed to 7.1% for the control group (p = 0.06). When compared with other chronic illnesses, the costs of patients with celiac disease were similar to those of patients with diabetes and hypertension.

**Conclusions:**

Patients with Celiac disease utilize medical services more than the general population. This research suggests that the use of medical resources by patients with Celiac disease may be higher than previously thought.

## Background

Celiac disease (CD) is an inherited autoimmune disorder that is triggered by the ingestion of gluten [1] with a prevalence of approximately 0.5-1% of the population [2-6]. Currently, the only available treatment of the disease is a lifelong abstinence from gluten containing products. CD can present in a variety of different ways such as anemia or short stature without gastrointestinal involvement and may be missed by physicians. Lack of diagnosis and non-adherence to treatment may have a number of adverse health outcomes such as osteoporosis, increased occurrence of autoimmune diseases, decreased quality adjusted life years and increased standardized mortality ratio [7-10]. Furthermore, in poorer countries, children with CD may commonly present with chronic diarrhea and malnutrition, correct diagnosis is often overlooked, and may result in unnecessary mortality [11]. Thus CD can be regarded as a hidden public health problem both in the developed and developing world [12-14]. The costs of a particular disease in different countries will always be dependent of the structure of a particular healthcare system. For instance, access to secondary care, dieticians and the cost of gluten free food will be different in each system. Analysis from the United Kingdom shows that significant additional primary care costs are associated with CD [15]. Three studies, two American and one from the Mediterranean region have shown reduction in direct medical costs of selected services after diagnosis [13,16,17]. As a result of the increasing prevalence of CD, the public health burden of the disease makes it a possible candidate for universal screening [18-20]. One criterion that needs to be assessed in this debate is the cost to the health system of the disease. The aim of our study was to identify the direct costs of patients diagnosed with CD.

## Methods

Settings & participants: All residents of Israel are insured by one of four non-profit, integrated Preferred Provider Organizations (PPO). This study was conducted in Maccabi Healthcare Services (MHS), the second largest PPO in Israel insuring over two million members. According to Israeli National Health Insurance Act legislated in 1994, MHS is obliged to insure every citizen who wishes to join it, irrespectively to age, gender, physical condition or any other criterion. Therefore, every section in the Israeli population is well represented in MHS.

Sources of data: The MHS central data repository retains complete historical records of patient demographic data, physician data, laboratory results, and costs using the patient's unique national identification number. A computerized medical record (CMR) is used by all 3780 physicians caring for a population of two million individuals in over 4000 community clinics/surgeries. Patients have blood samples taken at one of the 260 collection points throughout the country and all laboratory testing is undertaken in one central location. There are no patient copayments for laboratory testing. The CMR runs locally on a personal computer located in the physician’s office and is used exclusively for the physicians' patients insured by MHS. It exchanges data with the central data repository using proprietary transactions sent by modem over telephone lines or through a wide area network. Laboratory test results, among other data, are downloaded on a daily basis and are reviewed by physicians at their leisure either on-screen or in printed form. After being accepted by the doctor, these lab reports become an integral part of the patient’s medical record. This study was approved by the PPO’s Institutional Review Board on 23.7.2006.

Cohort definition: This retrospective case control study covered the period 2003–2006. All patients that were identified with CD from this period were included in our cohort. We compared costs between patients with and without CD. The cohort with CD was defined by an ICD9 diagnosis in their CRM and a positive blood test for CD serology (tissue transglutaminase antibodies, Phadia, Germany). The control group from the rest of the MHS population was matched in clusters for gender and age on an 8:1 basis.

We compared costs by admission, medication purchase, laboratory, imaging (including computerized tomography, ultrasound and magnetic resonance imaging) and overall costs. The number of laboratory test were broken down by Hb, WBC, Bilirubin, Creatinine, Cholesterol, HDL, LDL, Triglyceride, AST, Urea, Uric Acid, Total T4, Free T4, TSH, IgA, IgG, IgM, IgE. In addition we compared the number of visits to primary and secondary care physicians as well as visits to dieticians.

We also examined the comorbidities of the two groups as follows: Oncology patients within five years of diagnosis or active treatment. Diabetes mellitus (DM), Ischemic heart disease (IHD), Myocardial infarction (MI), Atrial Fibrillation (AF), Congestive cardiac disease (CHF) Hypertension (HTN), and those patients defined by the health ministry as having selected severe illness which includes dialysis, AIDS, thalassemia major. We also compared costs after a logarithmic transformation.

Cost assessment: In the MHS accounting system every treatment is automatically converted into money terms according to real market prices. The databases allow all costs to be aggregated according to main cost-branches (hospital admission, emergency department visits, secondary care including gastroenterology care, primary care visits, medications, laboratory and imaging) and computed individually for each member.

CD-related cost assessment: The actual direct cost of a CD patient was assessed by comparing his/her cost during study period with the mean cost of all other MHS members, matched for age group and gender. No externalities or direct non-medical costs were included. Cost of gluten free nutrition was not included. All costs were computed in New Israeli Shekel (NIS).

Statistical analysis: Confidence intervals for patient and disease-related costs were computed by the independent sample T-test and Mann–Whitney U-test for continuous variables and Chi test or Fisher’s exact test for discrete variables. The analysis was done independently for each of the three years and as an average for the period 2003–6 for each index case and their controls. A linear step wise regression was performed for the independent variables such as age, gender and the presence of CD. The dependent variable was the log of costs rather than the actual costs because the costs were not distributed normally. The significance level for all comparisons was corrected by the false discovery rate (FDR) [21]. The analysis was undertaken using SPSS16 and MATLAB.

## Results

Our cohort of patients with celiac disease comprised 1,754 patients aged 25.7 ± 19.2 years with a control group of 15,040 patients aged 28.8 ± 26.4 years. The proportion of males in these two groups was 34% and 42% respectively (p < 0.001). The difference in age between the two groups was not significant (p = 0.15).

**Figure 1:**
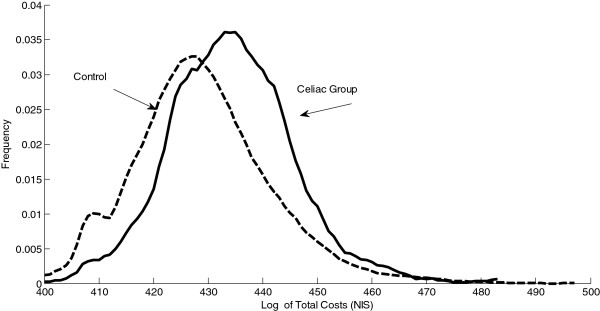
Log of total yearly costs for patients with celiac disease and control group.

The total costs of the patients with CD were significantly higher than that of the control group. The data is presented as a log value in order to approximate a standard distribution (Figure 1). Table 1 describes the costs by type of expenditure. We see that there is a significant difference between costs for all types of expenditure with the exception of MRI scans. For the years 2005–2006 the rate of hospital admission was 7.98% as opposed to 7.1% for the control group (p = 0.06). (2003/4 data for hospital admissions was incomplete). Using the FDR algorithm a p value less than 0.003 is considered significant [21].

**Table 1:** Costs by type of expenditure between groups

**Group statistics**					
**Costs**		**N**	**Mean**	**Std. deviation**	**p-value**
Log HOSPITAL costs	Control	15,039	.5361	1.22208	0.012
	Celiac	1,754	.6135	1.27476	
Log DRUGS costs	Control	15,040	1.8062	.90631	<0.0001
	Celiac	1,754	2.1738	.73001	
Log LAB costs	Control	15,040	1.5037	.93370	<0.0001
	Celiac	1,754	2.1996	.51023	
Log standard radiology costs	Control	15,040	.9713	.97449	<0.0001
	Celiac	1,754	1.3291	.94472	
Log CT costs	Control	15,040	.1105	.49551	<0.001
	Celiac	1,754	.1986	.64724	
Log MRI costs	Control	15,040	.0363	.32318	0.20
	Celiac	1,754	.0481	.37254	
Log Total costs	Control	14,997	2.7926	.82677	<0.0001
	Celiac	1,754	3.1865	.49778	

When examined for use of imaging, a significant difference between groups was found only for ultrasound. 41% for the celiac patients compared to 35.7% for the rest (p < 0.001). Utilization of primary and secondary care showed a statistical difference between groups for visits to gastroenterologists 1.06 and 0.23 visits per year (p < 0.001) respectively and medical nutritionists 0.5 and 0.24 visits per year (p < 0.001) respectively. Endoscopy costs fall under two separate categories in Table 2; “Hospital costs” and “Other costs” depending on where the procedure was undertaken. The rate of laboratory testing for the celiac patients was significantly greater for every category examined; Hematology Chemistry and Immunology. The FDR was less than 0.00115.

**Table 2:** Stepwise linear regression of variables by log of total costs

**Coefficients**^**a**^						
**Model**		**Unstandardized coefficients**		**Standardized coefficients**	**t**	**Sig.**
		**B**	**Std. error**	**Beta**		
1	(Constant)	2.696	.012		229.649	.000
	Celiac Group	.401	.019	.152	20.901	.000
	Patient Age	.002	.000	.063	7.200	.000
	Gender (male)	-.091	.012	-.056	-7.605	.000
	MI	.472	.049	.076	9.611	.000
	CHF	.347	.104	.025	3.326	.001
	Cancer	.639	.046	.102	13.749	.000
	Hypertension	.416	.025	.150	16.517	.000
	Special category	1.718	.380	.033	4.520	.000
	Diabetes	.310	.037	.067	8.441	.000

^a^Dependent Variable: log Total Costs.

When we examined for comorbidity between the groups by performing logarithmic regression we found that the celiac patients had Odds Ratios of 1.073 (95% CI 1.014-1.136) and 1.055 (95% CI 0.998-1.116) for Cardiac disease and Diabetes Mellitus respectively. When we examined the costs between groups using stepwise logarithmic regression and the log of total costs as the dependent variable, (Table 2) we see that that celiac patients have substantially increased costs compared to that of the control group. Interestingly the costs of patients with celiac were similar to those of patients with diabetes and hypertension.

## Discussion

The clear finding from our study is that patients with CD utilize medical services more than general population in most categories of expenditure examined. This is somewhat surprising in view of the fact that many of these patients will be asymptomatic and observing their gluten free diet. Our null hypothesis was that diagnosed patients would have costs that were similar to the control group. Previous studies have shown a decrease in medical expenditure after the initiation of treatment. A 30% reduction in direct medical costs after diagnosis of CD was reported by Green [13]; the mean medical expenditure decreased from $8502 per capita to $7133 for the 2 years after diagnosis of CD. A study by Long et al. showed that the annual medical cost in the year preceding the diagnosis of CD, excluding diagnostic costs, was estimated to be $5023/patient, $1764 more than the cost of the same patients in the year after diagnosis. In the four years preceding the diagnosis of symptomatic CD, the direct medical cost was estimated to be $11 037/patient. For age-matched control individual, not affected by CD, the cost over 4 years was estimated at $7073, with a difference of $3964 (about $1000/patient per year). This difference was due to increased in-patients admissions, out-patient cost, laboratory tests, radiology, and office visits [17].

By our study design, it is impossible to prove or refute previous findings suggesting a decline in medical costs after diagnosis. However, in our study, where all patients already had a diagnosis of CD at baseline, there was no decrease in medical costs over the years. However it should be remembered that that most patients who have CD and adhere to their gluten free diets convert to negative serology. Thus this group would not be represented in our cohort and it may be that our cohort is a sicker population of patients.

When compared to the costs of other chronic disease states our sample shows similar costs to that of diabetes and hypertension. It should be remembered that the average age of our sample was under thirty years of age. This can be compared to an average age of 61.6 years for MHS patients with diabetes [22]. If our findings are replicated in other healthcare settings this will have significant implications for resource use in health systems since CD appears at a relatively young age, has a relatively high prevalence and long life expectancy.

Our study has a few limitations: the case definition of our CD group was made using the diagnosis and supporting laboratory data but was not validated by biopsy results. The lack of information in our study relating to time of diagnosis is a deficiency as we know that CD costs peak in proximity to the time of diagnosis and if for some reason there were a high number of diagnoses of new CD in close proximity to the years 2003–6 this could create bias. In addition, we do not know how many of the CD patients were adhering to their gluten free diets, and it could be that the increased medical costs were due to non-adherence and complications of CD. Also, costs of gluten free products were not included in our analysis, though this would increase the medical costs of CD, strengthening the refutation of our null hypothesis. Although all sections of the population are well represented in MHS, MHS has higher market penetration in the centre of the country which is both wealthier and with greater access to secondary care than the periphery. This may result in more costs than in the country’s periphery. Finally, we do not know the length of time from diagnosis of each patient, whether they were symptomatic or not and what was the impact of their disease on quality of life, information that would enable us to explore the factors related to the increased costs. The strength of our study is that our results are based on analysis of complete medical utilization costs and admission data with the exception of private consultations in a whole population depicting the medical costs in real life.

## Conclusion

As the incidence of CD is increasing in all countries, it becomes more important to assess the financial consequences of diagnosis. Our study suggests that the patients with CD require increased medical resources similar to patients who suffer from diabetes or hypertension, unrelated to the time of diagnosis.

## Competing interests

The authors declare that they have no conflict of interest. There has been no external funding for this study.

## Authors’ contributions

ADH and RS planned the study. RE and ADH drafted and reviewed the manuscript. ML was responsible for the statistical analysis. Each author has approved the final draft submitted.
